# Hydrogel Containing Biogenic Silver Nanoparticles and *Origanum vulgare* Essential Oil for Burn Wounds: Antimicrobial Efficacy Using Ex Vivo and In Vivo Methods Against Multidrug-Resistant Microorganisms

**DOI:** 10.3390/pharmaceutics17040503

**Published:** 2025-04-10

**Authors:** Angela Hitomi Kimura, Débora Dahmer, Luana Ayumi Isawa, Ana Beatriz Olivetti da Silva, Lucas Marcelino dos Santos Souza, Pedro Henrique Takata, Sara Scandorieiro, Anastácia Nikolaos Deonas, Jennifer Germiniani-Cardozo, Eliana Carolina Vespero, Marcia Regina Eches Perugini, Nilton Lincopan, Audrey Alesandra Stinghen Garcia Lonni, Gerson Nakazato, Renata Katsuko Takayama Kobayashi

**Affiliations:** 1 Laboratory of Basic and Applied Bacteriology, Department of Microbiology, Center of Biological Sciences, State University of Londrina, Londrina 86057-970, Brazil; angela.hkimura@uel.br (A.H.K.); luaayu.isawa22@uel.br (L.A.I.); ana.b.olivetti@uel.br (A.B.O.d.S.); lucas.marcelino1@uel.br (L.M.d.S.S.); pedrotakata2@gmail.com (P.H.T.); gnakazato@uel.br (G.N.); 2 Department of Biochemistry and Biotechnology, State University of Londrina, Londrina 86057-970, Brazil; deboradahmer.dd@uel.br (D.D.); anastacia.nikolaos@uel.br (A.N.D.); jennifer.germiniani@uel.br (J.G.-C.); 3 Laboratory of Innovation and Cosmeceutical Technology, Department of Pharmaceutical Sciences, Center of Health Sciences, University Hospital of Londrina, Londrina 86038-350, Brazil; sarascandorieiromicro@gmail.com (S.S.); audrey@uel.br (A.A.S.G.L.); 4 Department of Pathology, Clinical and Toxicological Analysis, Center of Health Sciences, University Hospital of Londrina, Londrina 86038-350, Brazil; elianacv@uel.br (E.C.V.); marciaperugini@uel.com (M.R.E.P.); 5 Department of Microbiology, Institute of Biomedical Sciences, University of São Paulo, São Paulo 05508-900, Brazil; lincopan@usp.br

**Keywords:** *Galleria mellonella*, porcine skin, *Staphylococcus aureus*, *Klebsiella pneumoniae*, *Pseudomonas aeruginosa*, green nanotechnology, oregano essential oil

## Abstract

**Background/Objectives:** Wounds from burns are susceptible to infections, allowing multidrug-resistant microorganisms to complicate treatments and patient recovery. This highlights the development of new strategies to control these microorganisms. This work evaluated the antibacterial activity of hydrogels containing biogenic silver nanoparticles (bio-AgNP) and *Origanum vulgare* essential oil (OEO) against multidrug-resistant bacteria. **Methods:** The formulations were subjected to organoleptic, pharmacotechnical, and stability characterization and antimicrobial activity assessment by time–kill tests and alternative methods, an ex vivo model using porcine skin, and an in vivo model using *Galleria mellonella*. **Results:** All hydrogels maintained their stability after the thermal stress. The hydrogel containing bio-AgNP + OEO 1% (HAgNP + OEO1) presented bactericidal effectiveness, within 2 h, against both Gram-positive and Gram-negative multidrug-resistant bacteria in the time–kill test. For alternative testing, HAgNP + OEO1 was compared with 1% silver sulfadiazine (SS) and the base formulation. In the ex vivo test, both HAgNP + OEO1 and SS treatments showed a similar reduction in superficial washing of the burn for *S. aureus* 999, while for *P. aeruginosa*, the reduction was more expressive for SS treatment. In the burn tissue, HAgNP + OEO1 treatment was more effective against *S. aureus* 999, while for *P. aeruginosa* 1461, both formulations were similarly effective. In the *Galleria mellonella* test, survival rates after 48 h were 84% for the control group (base) and 50% for both HAgNP + OEO1 and SS treatment groups. **Conclusions:** This study demonstrates that the hydrogel combining antimicrobials is effective against multidrug-resistant microorganisms, offering a promising alternative for the treatment of infected burns.

## 1. Introduction

The skin is the largest organ in the human body, covering the entire body surface and ensuring much of the relationship between the internal and external environment [1]. This organ represents 10 to 15% of body weight [2] and performs essential functions for human health, such as contributing to immune function, body thermoregulation, protection against pathogens and injuries, and perception of the environment through sensory nerves, among others [1,3]. The interface between the skin and the external environment results in the colonization of the surface, more specifically the epidermis, by fungi, bacteria, and viruses [4,5]. This set of microorganisms lives in microbial symbiosis and forms the healthy microbiota of the skin. Maintaining the integrity of the skin barrier is crucial for protection against pathogens, in addition to hindering invasion by foreign agents.

The skin is subject to external aggression, and one of the injuries it may sustain is that caused by burns. Burns can be caused by friction, direct contact with cold, heat, chemical, electrical, and radioactive agents [6]. The injuries result in partial or total destruction of the skin, with variations in size and depth.

In Brazil, the Ministry of Health published the results of a survey carried out between 2015 and 2020, which found that 19,772 deaths from burns occurred. Of this total, 53.3% (n = 10,545) were due to thermal burns; 46.1% (n = 9117) were due to electrical burns; and 0.6% (n = 110) were attributed to other burns (by chemical agents, frostbite, and radiation) [7]. The World Health Organization highlights that burns are a global public health problem and occur mostly in low- and middle-income countries. It is estimated that 180,000 deaths occur every year [8]. The complex nature of these injuries can have a significant impact on an individual’s mental health and quality of life. Therefore, studies in this area are essential to understand the impact that burns have on the health of the world’s population [9].

Once the skin barrier is compromised, wounds caused by burns can be infected with multidrug-resistant microorganisms, leading to complications and worsening of the patient’s clinical condition [10]. Burns cause the loss of the physical barrier function, allowing the invasion of microorganisms and the development of infections, which can evolve into more serious conditions, such as sepsis [11]. In addition, the dysregulation of the immune response that patients with extensive burns present is another complicating factor that increases the predisposition to infections [12,13].

After thermal injury, microorganisms in the epidermis have a temporary colonization decrease. Recolonization of a superficial lesion can occur through the surviving microbiota in the hair follicles and sebaceous glands [14]. In this initial phase, Gram-positive bacteria are usually found, including the species *Staphylococcus*, *Corynebacterium*, and *Streptococcus* [15,16]. In the second phase, 5–7 days after the injury, the site becomes predominantly colonized by Gram-negative microorganisms. The main microorganisms found include *Pseudomonas aeruginosa*, *Escherichia coli*, *Enterobacter* species, and *Proteus* [10,15]. In addition to this, methicillin-resistant *Staphylococcus aureus* (MRSA), *Klebsiella pneumoniae*, and multidrug-resistant *Acinetobacter* have also been identified in burns at this stage [10,14].

In superficial burns, tissue perfusion of the dermis is not impaired, which makes the development of an invasive infection less frequent [15]. On the contrary, deep burns are a favorable environment for microbial development. The presence of moist, protein-rich dead skin favors bacterial proliferation and invasion, which can result in wound infection [15,16]. Another challenge in wound treatment is the formation of biofilm [17]. This microbial community, surrounded by an extracellular polymeric matrix, acts as a protection, interfering with the action of antimicrobials, resulting in increased mortality in burn patients.

Studies report that *P. aeruginosa* and *S. aureus* are the microorganisms frequently found in burn injuries [18,19]. The indiscriminate use of antimicrobials can select multidrug-resistant strains and, consequently, patients become more susceptible to infections by these microorganisms, making treatment considerably more difficult. This highlights the importance of investing in the development of innovative approaches and alternative antimicrobials.

Antimicrobial resistance is a global health concern. In 2014, a study conducted by the British government estimated that by 2050, there would be 10 million deaths caused by multidrug-resistant microorganisms, exceeding the number of deaths caused by other diseases such as diabetes (1.5 million) and cancer (8.2 million) [20]. The growing resistance of microorganisms to available antimicrobials is one of the challenges of current medicine, and the spread of multidrug-resistant microorganisms has mobilized government entities to develop measures to combat this issue of global concern [20], such as the use of natural products and nanotechnology.

The antimicrobial action mechanisms of AgNPs is not fully understood, but reports in the literature indicate that this active, initially, adheres on the surface and plasma membrane, due to electrostatic attraction and affinity for sulfur proteins; after this, silver ions increase the permeability of the plasma membrane, resulting in the rupture of the bacterial wall; next, AgNPs and Ag^+^ are absorbed, which can deactivate respiratory enzymes and generate reactive oxygen species, in addition to free radicals; finally, damage to the intracellular machinery can lead to apoptosis. Ag^+^ ions interact with several structures of the cell, including DNA, which can cause problems in cell replication [21,22,23,24].

In the face of multidrug resistance, the development of resistance mechanisms may lead to limited treatment options. One strategy that has shown promise is the combined use of antimicrobials such as EOs with bio-AgNP [25,26]. In this context, the *Origanum vulgare* essential oil (oregano) can be a suitable choice. The antimicrobial activity of OEO is attributed to its secondary metabolites, including the major component carvacrol, besides thymol, *p*-cymene, γ-terpinene, and linalool [27]. This potential activity is evidenced in several studies, including against multidrug-resistant microorganisms. The research of Scandorieiro et al. (2016) [28] shows the effectiveness of OEO, carvacrol, and thymol against different microorganisms, including methicillin-resistant *S. aureus* (MRSA), extended-spectrum beta-lactamase (ESBL)-producing *E. coli*, and carbapenemase-producing *K. pneumoniae* (KPC) [28]. In addition to their antimicrobial properties, EOs also play an important role in wound treatment. Specifically, oils that contain phenolic compounds, such as carvacrol and thymol, have the ability to stimulate angiogenesis and the skin re-epithelialization process [29].

For the treatment of infected second- and third-degree burns, silver sulfadiazine is the most widely used topical antimicrobial [30]. It is available as a cream containing 1% silver sulfadiazine and has a broad spectrum of action, combating both Gram-negative and Gram-positive bacteria, in addition to being effective against fungi and yeasts [15,31]. Although it has been used for decades, silver sulfadiazine has some disadvantages, such as the formation of pseudo-eschar on the wound surface. This pseudo-eschar is a pasty, yellowish-white exudate that hinders the healing process, making mechanical debridement necessary to remove it [15,32].

Hydrogels can be used as an approach to cover the burning’s wounds, avoiding other injuries and the development of infections while maintaining a moist environment. A variety of polymers can be used for this purpose, such as Lecigel^®^. This material is a gelling agent composed of sodium acrylate copolymers and lecithin with emulsifying properties that have been used in a wide range of semi-solid formulations, such as gel, creams, gel-creams, and hydrogels. Some advantages of this polymer are viscosity, stability in a wide range of pHs, use in cold or hot processes, and the aggregation of a pleasant sensorial characteristic to the formulation, which includes cool, soft, and non-greasy effects [33]. Active ingredients intended for cutaneous application are improved when incorporated into a matrix suitable for topical application, that is, the development of a semi-solid formulation such as a hydrogel to increase the viscosity of the product, allowing adequate contact with the skin surface and the release of these active ingredients at the target site. Furthermore, the hydrogels are biocompatible and usually non-adhesive, being easily applied and/or removed from the wound, which allows their wide application in the medical field as a class representative of versatile polymeric biomaterials that can incorporate antimicrobial actives, including nanoparticles and essential oils, for the treatment of varied conditions, such as wounds [34].

In this way, the aim of this work was to develop hydrogels containing bio-AgNP and OEO for the treatment of wounds caused by burns and to evaluate their antibacterial activity against multi-resistant Gram-negative and Gram-positive bacteria. In view of the multi-resistant microorganisms problem, research and development of alternative strategies to control multidrug-resistant microorganisms are extremely necessary, and biogenic silver nanoparticles (bio-AgNP) and oregano essential oil (OEO) are presented as alternatives to combat this multidrug resistance.

For the evaluation of antimicrobial activity, alternative methods were used, including an ex vivo model using porcine skin and an in vivo model using *Galleria mellonella* larvae. As is well known, animals have been used for a long time in drug testing, efficacy validation, and safety evaluation, being the major contributors to clinical research. As a result, millions of animals are euthanized every year. Due to ethical and animal welfare debates, the need for new alternatives to reduce and decrease the use of animals in scientific studies is essential [35]. The use of invertebrates such as *Galleria mellonella*, for example, is a new approach in the bacteriological field for antibacterial drug research and presents some advantages, such as the use of several larvae in a single experiment, no ethical concerns, and economic benefits [36]. In addition, *G. mellonella* can be used as an invertebrate model of burn and wound infection, according to the study developed by Maslova et al. (2020) [37], as an alternative to the mouse model. Another alternative includes the use of ex vivo models with animal tissues, such as porcine skin, which has been reported in some studies about burn wounds [38,39]. Some advantages of this model are the similarities of porcine skin with human skin, the availability and low cost of this tissue, and more controlled experimental conditions than in vivo [40].

## 2. Materials and Methods

### 2.1. Bacterial Isolates

The bacteria used in this study were isolated from clinical samples of patients at the University Hospital of Londrina, Brazil, and the Institute of Biomedical Sciences (ICB) of the University of São Paulo, Brazil. The clinical isolates were *P. aeruginosa* 117, *P. aeruginosa* 1634, *P. aeruginosa* 1461, *P. aeruginosa* 2491, *P. aeruginosa* 2815, penicillin-sensitive *S. aureus* (PRSA) 207, PRSA 521, *K. pneumoniae* 11034, and *K. pneumoniae* 10797. The reference strains were *P. aeruginosa* ATCC 27853, methicillin-resistant *S. aureus* N315, and *Klebsiella pneumoniae* ATCC 700603. These microorganisms were cultured in Mueller–Hinton broth (MHB) and stored at −20 °C in brain–heart infusion broth (BHI, Merck^®^, Darmstadt, German) with 25% (*v*/*v*) glycerol (Merck^®^, Darmstadt, German).

### 2.2. Antimicrobial Sensitivity Profile

The antimicrobial sensitivity test was determined using the disk diffusion method, as described by the Clinical and Laboratory Standards Institute (CLSI) [41,42]. The following antibiotics were used: clindamycin (2 μg); oxacillin, ciprofloxacin, levofloxacin, (5 μg); cefpodoxime, tobramycin, ertapenem, imipenem, meropenem, norfloxacin, penicillin and gentamycin (10 μg); azithromycin, quinupristin-dalfopristin, tigecycline, erythromycin (15 μg); ampicillin-sulbactam (20 μg); cefazolin, cefepime, cefotaxime, ceftazidime, ceftriaxone, cefuroxime, cefoxitin, tetracycline, linezolid, amoxicillin-clavulanic acid, aztreonam, amikacin, nalidixic acid and chloramphenicol (30 μg); trimethoprim-sulfamethoxazole (1.25/23.75 μg); and piperacillin-tazobactam (110 μg), sulfonamide (300 μg) (Oxoid Ltd., Basingstoke, Hants, UK).

### 2.3. Antimicrobial Compounds

Oregano (*Origanum vulgare)* essential oil (OEO) was obtained from Ferquima (São Paulo, Brazil). According to the technical report, the *Origanum vulgare* leaves were processed by steam distillation, and the following components were described: carvacrol (72%), thymol (2%), γ-terpinene (4.8%), paracymene (5%), linalool (3%), and β-caryophyllene (3%). The density of the essential oil is 0.9468 g/mL.

Biogenic silver nanoparticles (bio-AgNP) were obtained from GRAL Bioativos (Nano VerdeAg^®^, Londrina, Brazil). The Bio-AgNP were synthesized from plant extract biomass, a process that was patented (BR 102021016375-5). The diameter of the nanoparticles and the zeta potential (ζ) were determined by the Litesizer DLS 500 (Anton Paar, Graz, Austria).

Silver sulfadiazine cream 1% (SS) (Dermazine^®^, Cristália, Brazil) was obtained from a commercial source.

### 2.4. In Vitro Analysis of the Antibacterial Activity of OEO and Bio-AgNP

The minimum inhibitory concentration (MIC) of the isolated antimicrobial agents was determined by the broth microdilution method according to CLSI [43]. The concentration range of the antimicrobials used in the tests were (i) 9.5–0.07 mg/mL for OEO and (ii) 8493.5–132.71 mg/mL for bio-AgNP. OEO was diluted in DMSO (dimethyl sulfoxide). The bacterial inoculum was prepared in phosphate-buffered saline (PBS 0.1 M, pH 7.2) and adjusted to the 0.5 MacFarland scale, which corresponds to 1.5 × 10^8^ CFU/mL. After 24 h of incubation at 37 °C, the MIC was determined as the lowest concentration capable of inhibiting visible microbial growth.

### 2.5. Antimicrobial Combination Assay

The interaction between the combination of OEO and bio-AgNP was determined by the methodology described by Scandorieiro et al. (2016) [28]. The antibacterial interaction between the actives was determined by the fractional inhibitory concentration index (FICI), according to Chin, Weitzman, and Della-Latta (1997) [44]. The determination of the index followed the Equation (1), by adding the Fractional Inhibitory Concentration (FIC—Equation (2)) of the actives.

(1) $$FICI={FIC}_{OEO}+{FIC}_{bio-AgNP}$$

where

(2) $$FIC=\frac{{MIC}_{combination}}{{MIC}_{individual}}$$

The FICI is interpreted as “synergistic” when ≤0.5; as “additive” when >0.5 and ≤1; as “indifferent” (no interaction) when >1 and <4; and as “antagonist” when ≥4.

### 2.6. Hydrogel Development

To evaluate the antimicrobial activity of the active ingredients alone and in combination, six different hydrogels were developed: hydrogel without antimicrobial agent (base); hydrogel containing bio-AgNP 500 µM (HAgNP); hydrogel containing OEO 1% (HOEO1); hydrogel containing OEO 0.5% (HOEO0.5); hydrogel containing bio-AgNP 500 µM + OEO 1% (HAgNP + OEO1) and hydrogel containing bio-AgNP 500 µM + OEO 0.5% (HAgNP + OEO0.5). The base was considered the negative control.

The gelling agent used was Lecigel™ (Sodium Acrylates Copolymer (and) Lecithin), manufactured by Lucas Meyer Cosmetics (Massy, France). The hydrogels were prepared as follows: initially, the gelling agent was dispersed in purified water under constant mechanical stirring (600 rpm) in a mechanical stirrer (Fisatom, São Paulo, Brazil). Then, glycerin, propylene glycol, butylated hydroxytoluene, caprylic and capric acid triglycerides, and phenoxyethanol and methylisothiazolinone were added under stirring until complete homogenization. Finally, the antimicrobial agents (bio-AgNP 500 µM, OEO 1% *v*/*v*, and OEO 0.5% *v*/*v*) were added to the respective hydrogels. The pH was adjusted between 6.9 and 7.2, which corresponds to the pH of the wounds [45]. The formulations were packaged in opaque polyethylene (PP) plastic bottles and kept refrigerated for 24 h for subsequent tests.

### 2.7. Pharmacotechnical Characterization of Hydrogels

#### 2.7.1. Pre-Stability Test

The centrifugation test was carried out as described in the Cosmetics Stability Guide [46]. In triplicate, 5 g of hydrogel was added to conical tubes and centrifuged (Baby I Fanem 206-BL centrifuge, Guarulhos, Brazil) for 30 min at 3000 rpm at room temperature.

#### 2.7.2. Organoleptic Characterization

The appearance, color, and odor of the hydrogels were analyzed according to the Cosmetic Products Quality Control Guide [47] and determined as N = normal (no change); LM = slightly modified; M = modified; and IM = intensely modified [46].

#### 2.7.3. Physical–Chemical Evaluation

The pH and density of the hydrogels were analyzed according to the Cosmetic Products Quality Control Guide [47]. To determine the pH, the electrode (PG 1800, Gehaka, São Paulo, Brazil) was inserted directly into the hydrogels, in triplicate, after the calibration. The density of the hydrogels was determined using a pycnometer (PHOX) with a capacity of 10 mL. Firstly, the empty weight of the pycnometer was determined on an analytical scale (Marte Científica, São Paulo, Brazil). Then, purified water was added, and its weight was determined again (in triplicate). Finally, the same process was performed for the hydrogels, which were previously diluted in purified water in a ratio of 1:10. The density calculation was carried out according to the Equation (3):

(3) $$d=\frac{M_{1}-M_{0}}{M_{2}-M_{0}}$$

where

M_0_: mass of the empty pycnometer;
M_1_: mass of the pycnometer with the sample diluted in purified water;
M_2_: mass of the pycnometer with pure water.

#### 2.7.4. Spreadability

The spreadability assessment was performed according to the parallel plate method [48]. A glass plate (20 × 20) with a millimeter scale positioned below it was used. Then, one gram of the hydrogels was added to the middle of this plate, and another glass plate with known weight was placed under the formulation. After 1 min, the sample diameter was measured in millimeters. Then, a weight of 2 g was added under the sample, and the diameter was determined after 1 min. The same procedure was performed for the weights of 5 g and 10 g, respectively. The test was performed in triplicate, and the results were expressed according to the Equation (4):

(4) $$Ei=\frac{d^{2}\cdot \pi }{4}$$

where

Ei: spreadability of the sample for a given weight i (mm^2^);
d: average diameter (mm).

#### 2.7.5. Preliminary Stability Test

The preliminary stability test was carried out in accordance with the Cosmetic Product Stability Guide [46]. Forty grams of hydrogel were weighed in duplicate and packed in polypropylene bottles. Each duplicate was subjected to different conditions for 15 days: (i) in a cupboard, protected from light and at room temperature (25 °C ± 3 °C); (ii) 24 h cycles at 5 °C ± 2 °C and 40 °C ± 2 °C [46]. The hydrogels were analyzed for organoleptic characteristics, pH, density, and spreadability on day 1 (T0) and after 15 days (T15) of thermal stress. The hydrogels stored at room temperature were taken as a reference for comparison purposes.

### 2.8. Evaluation of In Vitro Antimicrobial Activity

The time–kill assay was performed according to the National Committee on Clinical Laboratory Standards (NCCLS) [49], with modifications. Microorganisms of each bacterial species were selected considering their resistance profile. The microorganisms stored in BHI glycerol were cultured in tryptone soy broth (TSB—Difco^®^) for 24 h and subsequently plated on tryptone soy agar (TSA—Difco^®^) for 24 h. The bacterial inoculum was prepared in PBS and adjusted to the 0.5 MacFarland scale, which corresponds to approximately 1.5 × 10^8^ CFU/mL. Then, 1 g of each formulation (base; HAgNP; HOEO1; HOEO0.5; HAgNP + OEO1; HAgNP + OEO0.5 and silver sulfadiazine 1%) was added to conical tubes with 9 mL of PBS (dilution 1:10). Then, 100 µL of the bacterial inoculum was added and serial dilution of the initial time (T0) was performed. For this, 100 µL of this sample was added to 900 µL of PBS, and 10 µL (in triplicate) was placed in TSA. The plates were incubated for 24 h at 37 °C. The formulations were incubated at 37 °C, and serial dilution was performed at the determined times (2 h, 8 h, and 24 h).

### 2.9. Evaluation of Antimicrobial Activity in an Ex Vivo Model

#### 2.9.1. Preparation of the Porcine Skin

The porcine skin was obtained from a butcher in Londrina, Brazil. Initially, the porcine skin was sanitized with neutral detergent, and the material was packed in aluminum foil and plastic bags and stored at −20 °C. For the ex vivo tests, the porcine skin was kept at room temperature in a sterilized Petri dish for 20 min. After this time, the porcine skin was subjected to the disinfection process, carried out according to the methodology described by Sousa et al. (2023) [50].

#### 2.9.2. Treatment with Antimicrobial Formulation on Porcine Skin

The procedure for burning and adding the bacteria to the lesion was carried out according to Andersson et al. (2021) [40], with modifications. The burns on the porcine skin were made with a 6 mm diameter round iron tip attached to a soldering iron (FAME, Brazil), previously heated to approximately 140 °C. The temperature was monitored with a Jiaxi digital laser thermometer (model 33207—China). The tip was sanitized with 70% alcohol and held vertically on the surface of the porcine skin for approximately 40 s. Next, 10 µL of bacterial inoculum was added, corresponding to 0.5 on the McFarland scale. The porcine skin was incubated for 2 h at 37 °C to establish the infection. After that, each lesion was completely covered with 0.1 g of the formulation (base; HAgNP + OEO1; and 1% silver sulfadiazine). All the porcine skins were incubated for 5 h at 37 °C.

#### 2.9.3. Viable Cell Count

After the incubation time, the viable cells were quantified using two independent procedures: (1) topical washing of the burn lesions and (2) analysis of samples from the topical washing. In the first procedure, each lesion was washed with 40 µL of PBS, and 6 mm diameter stainless steel cylinders were used to delimit the wash site. The resulting liquid was serially diluted (1:100) in PBS, and 10 µL was placed on plates containing specific culture media: salted mannitol agar (Kasvi, Madri, Spain) for Gram-positives and MacConkey agar (Kasvi) for Gram-negatives. These plates were incubated for 24 h at 37 °C. In the second procedure, all uninjured skin was removed with a disposable scalpel, and the resulting tissue was minced and transferred to microtubes containing 500 µL of PBS. The samples were homogenized in a vortex mixer (Scilogex SCI-VS, Rocky Hill, CT, USA) for 10 min. Serial dilution, plating, and incubation time followed the same process as described above.

### 2.10. Evaluation of Antimicrobial Activity in an In Vivo Model

#### 2.10.1. Management of Larvae

For the rearing of *Galleria mellonella* larvae, sterilized glass jars with a lid (center composed of a thin steel wire mesh) were used to allow ventilation [51]. The jars were filled with an artificial diet (4–5 cm high; composition: 416 g liquid honey, 200 g beeswax, 96 g powder milk, 188 g dried beer yeast, 385 g cornmeal, 160 g soy, 300 mL water, 300 mL glycerin, and 1.5 mL formaldehyde) [36] and placed during 5–6 weeks into a BOD incubator, until the larvae reached the 6th instar stage. Three times a week, the silk produced by the larvae was removed to prevent them from transforming into moths. Only cream-colored larvae with no injury marks were used for the tests.

#### 2.10.2. Antimicrobial Assessment

The hydrogels were evaluated for antimicrobial efficacy by in vivo tests according to the methodology described by Maslova et al. (2020) [37,52], with modifications; 1% silver sulfadiazine cream was used as a reference.

Fifty *Galleria mellonella* larvae, weighing 0.25–0.3 g, were selected and randomly distributed into five groups (bacterial control; burn control; base; HAgNP + OEO1; and 1% silver sulfadiazine) with ten larvae each. The first two groups corresponded to control with bacterial infection and without infection, and the last three were the treatment groups.

A steel tip with a 0.2 mm head was heated on a Bunsen burner and applied to the back of the larvae for approximately 2 s. After 10 min, 10 µL of the bacterial inoculum was applied to the burn. For the inoculum, bacterial colonies of *P. aeruginosa* 1461 were suspended in PBS and adjusted to a turbidity equivalent to 1.5 × 10^8^ CFU/mL. The formulations were applied after 10 min in the treatment groups, completely covering the lesion. The larvae were then incubated for 24 h at 37 °C.

The following day, larvae that did not respond to touch or that presented complete melanization (darkening) were discarded. The formulations were reapplied to the lesions, and the larvae were incubated again in the same conditions.

### 2.11. Statistical Analysis

Data analyses were performed using GraphPad Prism version 9.5.0 for Windows (GraphPad Software, San Diego, CA, USA). The two-way ANOVA test was used to analyze the time–kill curve, followed by Tukey’s post hoc test. For the ex vivo infection model, the one-way ANOVA test followed by Tukey’s post hoc test was used. A *p*-value ≤ 0.05 was considered statistically significant. The data from the in vivo assay were used for Kaplan–Meier survival curves, and the Log Rank test with *p* ≤ 0.05 was considered statistically significant.

## 3. Results

### 3.1. Antimicrobial Sensitivity Profile

The clinical isolates presented a resistance profile to different classes of antimicrobials, as indicated in Table 1. The isolates presented resistance to several classes of antimicrobials (penicillins, carbapenems, fluoroquinolones, cephalosporins, tetracycline, streptogramin, lincosamide, and aminoglycosides). The microorganisms were classified as MDR (multidrug-resistant) when they presented non-susceptibility to more than one antimicrobial in three different categories [53]. Furthermore, *Klebsiella pneumoniae* and *Pseudomonas aeruginosa* showed resistance to third-generation cephalosporins (cefotaxime, ceftriaxone, and ceftazidime) and carbapenems (ertapenem, meropenem, and imipenem) and were classified as MDR, KPC, and CR, respectively. *Staphylococcus aureus* was classified as MRSA (methicillin-resistant Staphylococcus aureus) as it was resistant to oxacillin.

### 3.2. Antimicrobial Combination Test

The antimicrobial compounds (OEO and bio-AgNP) were evaluated for minimum inhibitory concentration (MIC) as isolates and in combination, and the results are described in Table 2. In combination, both actives showed additive interaction for nine of the bacteria tested, and for five clinical isolates of *P. aeruginosa*, the interaction was synergistic.

### 3.3. Development of Hydrogels

**Figure 1:**
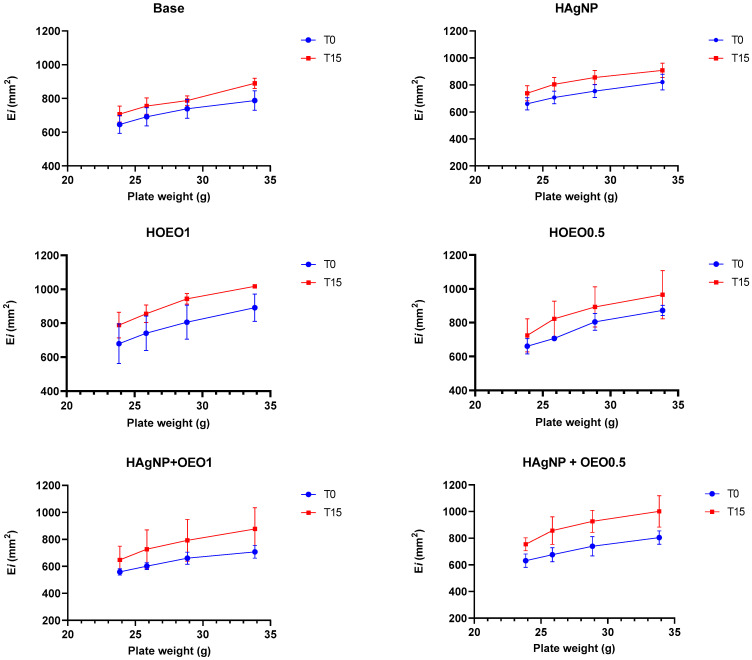
Spreadability of hydrogels at the preliminary stability test. Ei: spreadability of the sample for a given weight I; mm^2^: square millimeters; T0: zero time; T15: after 15 days of thermal stress; Base: formulation without actives; HAgNP: hydrogel containing bio-AgNP 500 µM; HO1: hydrogel containing OEO 1%; HO0.5: hydrogel containing OEO 0.5%; HAgNP + OEO1: hydrogel containing bio-AgNP 500 µM + OEO 1%; HAgNP + OEO0.5: hydrogel containing bio-AgNP 500 µM + OEO 0.5%.

After the pre-stability test, all hydrogels remained stable, without any visible change in color, odor, or aspect, and with no signs of phase separation. Regarding the organoleptic characteristics, base, HO1, and HO0.5 formulations presented a white color and were homogeneous, while for odor, the base was odorless, and HO1 and HO0.5 presented a smell characteristic of the OEO. Moreover, HAgNP, HAgNP + OEO1, and HAgNP + OEO0.5 formulations presented a light brown color (caramel) and were homogeneous, while for odor, HAgNP was odorless, and HAgNP + OEO1 and HAgNP + OEO0.5 presented a smell characteristic of the OEO. Related to the physical–chemical characteristics, all formulations presented a pH of around 6.9 and a density closer to 10.0. The spreadability of hydrogels (Figure 1) increased proportionally to the weights applied under the formulations, which was already expected.

The results of the preliminary stability of hydrogels are described in Table 3. For all analyses, the hydrogel in the initial state (T0) was considered as a comparison reference. All formulations did not change their initial characteristics, except for the color of the hydrogels containing bio-AgNP (HAgNP, HAgNP + OEO1, and HAgNP + OEO0.5), which showed a slight change at T15, as well as the results of pH and density, described in Table 4, and that likewise showed few variations between T0 and T15.

### 3.4. Evaluation of Antimicrobial Activity In Vitro

**Figure 2:**
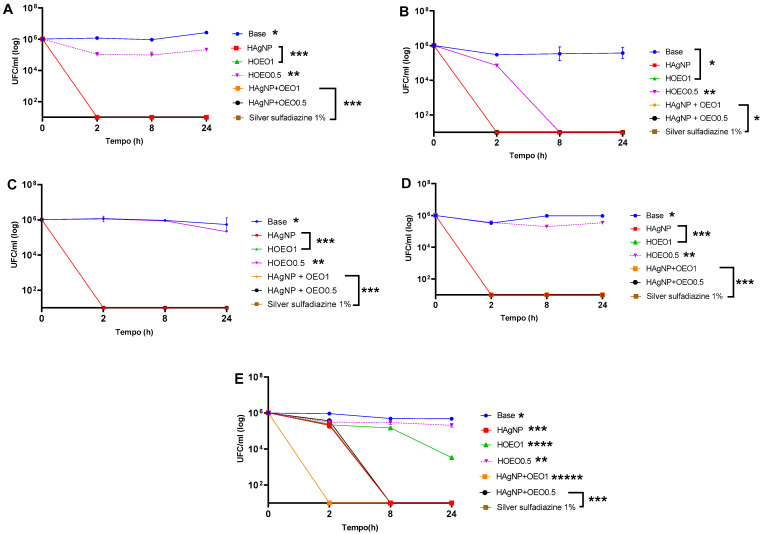
Time–kill curve of clinical isolates: (**A**) *P. aeruginosa* 1461; (**B**) *P. aeruginosa* 1634; (**C**) *P. aeruginosa* 117; (**D**) *K. pneumoniae* 11091; (**E**) *S. aureus* 999. Each isolate presented the *p*-value at different times: (**A**) * Base: 0–24 h; 2–8 h; 2–24 h; 8–24 h: *p* < 0.0001; ** HOEO0.5: 0–2 h; 0–8 h; 0–24 h; 2 -24 h; 8–24 h: *p* < 0.0001; *** HAgNP; HOEO1; HAgNP + OEO0.5; HAgNP + OEO1; silver sulfadiazine 1%: 0–2 h; 0–8 h; 0–24 h: *p* < 0.0001; (**B**) * Base; HAgNP; HOEO1; HAgNP + OEO0.5; HAgNP + OEO1; silver sulfadiazine 1%: 0–2 h; 0–8 h; 0–24 h: *p* < 0.0001; ** HOEO0.5: 0–2 h; 0–8 h; 0–24 h; 2–8 h; 2–24 h: *p* < 0.0001; (**C**) * Base: 0–24 h; 2–8 h; 2–24 h; 8–24 h: *p* < 0.0001; ** HOEO0.5: 0–24 h; 2–24 h; 8–24 h: *p* < 0.0001; *** HAgNP; HOEO1; HAgNP + OEO0.5; HAgNP + OEO1; silver sulfadiazine 1%: 0–2 h; 0–8 h; 0–24 h: *p* < 0.0001; (**D**) * Base: 0–2 h; 0–8 h; 0–24 h; 2–8 h; 2–24 h: *p* < 0.0001; ** HOEO0.5: 0–2 h; 0–8 h; 0–24 h; 2–8 h; 8–24 h: *p* < 0.0001; *** HAgNP; HOEO1; HAgNP + OEO0.5; HAgNP + OEO1; silver sulfadiazine 1%: 0–2 h; 0–8 h; 0–24 h: *p* < 0.0001; (**E**) * Base: 0–8 h; 0–24 h; 2–8 h; 2–24 h: *p* < 0.0001; ** HOEO0.5: 0–2 h; 0–8 h; 0–24 h; 2–24 h; 8–24 h: *p* < 0.0001; *** HAgNP; HAgNP + OEO0.5; silver sulfadiazine 1%: 0–2 h; 0–8 h; 0–24 h; 2–8 h; 2–24 h: *p* < 0.0001; **** HOEO1: 0–2 h; 0–8 h; 0–24 h; 2–8 h; 2–24 h; 8–24 h: *p* < 0.0001; ***** HAgNP + OEO1: 0–2 h; 0–8 h; 0–24 h: *p* < 0.0001.

The time–kill kinetics assay helps to understand the interactions between microorganisms and antimicrobial agents. The results are shown in Figure 2. The negative control (base) does not contain antimicrobial compounds and, therefore, there is no significant decrease in microbial growth over time.

The formulations HAgNP, HAgNP + OEO1, HAgNP + OEO0.5, and 1% silver sulfadiazine showed bactericidal activity after 2 h against *P. aeruginosa* 1461, *P. aeruginosa* 1634, *P. aeruginosa* 117, and *K. pneumoniae* 11091 (Figure 2A–D). The HO0.5 hydrogel maintained the CFU/ml level practically constant for 24 h, indicating a possible bacteriostatic activity.

For *S. aureus* 999, the HAgNP + OEO1 hydrogel showed bactericidal activity after 2 h (Figure 2E). The other formulations (HAgNP, HAgNP + OEO0.5, and silver sulfadiazine 1%) showed bactericidal action after 8 h. Unlike Gram-negative bacteria, HOEO1 did not show bactericidal activity; however, there was a reduction in the bacterial load after 8 h, demonstrating bacteriostatic activity. Similarly to Gram-negative bacteria, HOEO0.5 did not show a significant reduction in the microbial load over the 24 h period. In the statistical analysis, the difference was significant (*p* ≤ 0.05) for the treatments HAgNP, HAgNP + OEO1, HAgNP + OEO0.5, and silver sulfadiazine 1% for *P. aeruginosa* 1461, *P. aeruginosa* 1634, *P. aeruginosa* 117, *K. pneumoniae* 11091, and *S. aureus* 999.

### 3.5. Ex Vivo Model Analysis

**Figure 3:**
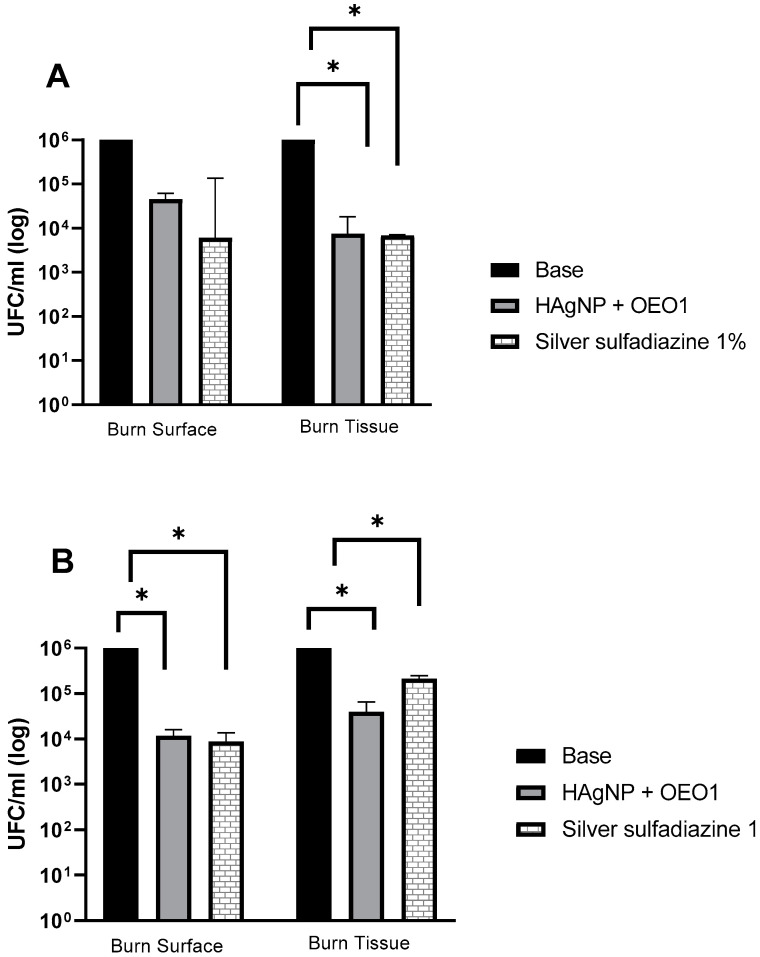
Evaluation of antimicrobial treatments in the ex vivo model. (**A**) *P. aeruginosa* 1461 and (**B**) *S. aureus* 999. Treatments that presented a value of *p* ≤ 0.005 are indicated by the symbol (*). Base: hydrogel without the actives; HAgNP + OEO1: hydrogel containing bio-AgNP 500 µM + OEO 1%.

The formulations were evaluated for their antimicrobial activity in an ex vivo model, and the results are shown in Figure 3. The “base” hydrogel was considered the negative control, and for statistical analysis, it was compared with the other treatments (HAgNP + OEO1 and 1% silver sulfadiazine). The *p*-value was ≤ 0.05, and, therefore, it was considered that there was a statistically significant difference for the treatment groups of *P. aeruginosa* 1461 and *S. aureus* 999.

The microbial analysis occurred at two moments of the test: first, when washing the burn surface, and then when recovering the microorganisms that may have invaded the tissue. In the surface wash, HAgNP + OEO1 and silver sulfadiazine 1% showed a reduction in the microorganism count (from 10^6^ to 10^4^) CFU/mL for *S. aureus* 999. For *P. aeruginosa* 1461, in both treatments (HAgNP + OEO1 and silver sulfadiazine 1%), the reduction was from log 6.0 to log 4.6 and log 3.9, respectively. In the burn tissue, the formulations containing HAgNP + OEO1 and 1% silver sulfadiazine reduced from log 6.0 to 4.60 and 5.33, respectively, for *S. aureus* 999. For *P. aeruginosa* 1461, both formulations reduced from 10^6^ to 10^4^.

In the statistical analysis, *P. aeruginosa* 1461 showed a significant difference (*p* ≤ 0.005) in the treatments between the base and HAgNP + OEO1 and 1% silver sulfadiazine in the burn tissue (Figure 3A). On the burn surface, there was no significant difference (*p* ≥ 0.005) between the base hydrogel and the other treatments. For *S. aureus* 999, the difference was significant (*p* ≤ 0.005) between the base hydrogel and HAgNP + OEO1 and 1% silver sulfadiazine both on the surface and in the burn tissue (Figure 3B).

### 3.6. In Vivo Model Analysis

**Figure 4:**
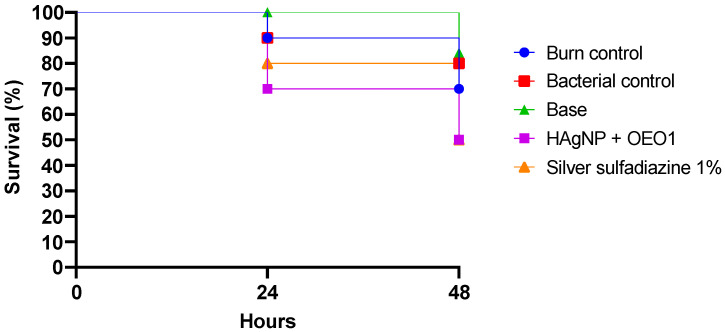
Survival curve in the in vivo model. *Galleria mellonella* was infected with *P. aeruginosa* 1461 and treated with the antimicrobial hydrogel containing bio-AgNP 500 µM + OEO 1% (HAgNP + OEO1). Log-rank *p*-value 0.1386. Base: hydrogel without the actives; Bacterial control: bacteria with saline (PBS 0.1 M, pH 7.2).

**Figure 5:**
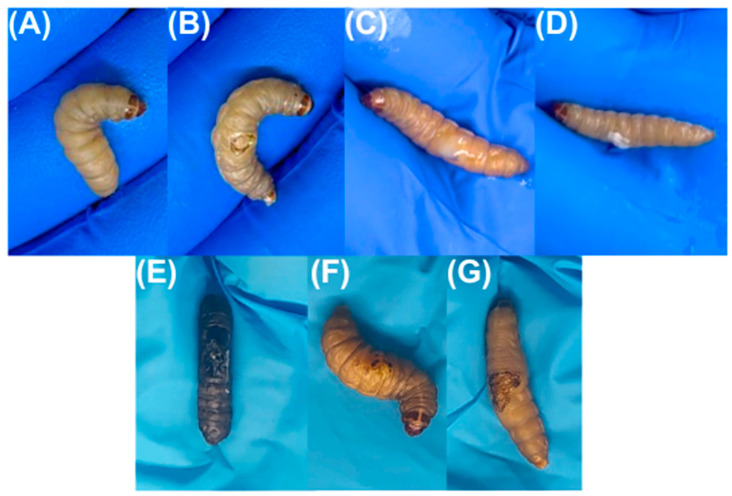
Larvae during the healing process. Figures (**A**–**D**) are related to time zero, that is, burning, infection, and treatment at the first time point, while Figures (**E**–**G**) represent the larvae after 48 h (end of the experiment). (**A**) Larvae before burning; (**B**) Larvae after burning and infection; (**C**) Larvae infected and after application of HAgNP + OEO1 formulation; (**D**) Larvae infected and after application of SS; (**E**) Larvae infected and without treatment; (**F**) Larvae infected and after treatment with HAgNP + OEO1, during the healing process; (**G**) Larvae infected and after treatment with SS, during the healing process.

*Galleria mellonella* showed the following survival rates after 24 h: 100% for the control group (base), 90% for the burn and bacteria control groups, 80% for 1% silver sulfadiazine, and 70% for the HAgNP + OEO1 treatment. At 48 h, the survival rates were 84% for the control group (base), 80% for the bacteria control group, 70% for the burn control group, and 50% for both treatments (HAgNP + OEO1 and 1% silver sulfadiazine) (Figure 4). The survival curves showed no significant difference (Log rank, *p* < 0.1386). Figure 5 illustrates the larvae and the different stages during the healing process.

## 4. Discussion

In this work, the actives bio-AgNP and OEO were evaluated for their antimicrobial activity, as isolates and in combination, as shown in Table 2, due to their potential alternatives as antimicrobials against Gram-negative and Gram-positive multidrug-resistant microorganisms. In a study developed by Saeki et al. (2021) [54], bio-AgNP synthesized by the reduction of silver nitrate, catalyzed by an enzyme preparation from *Fusarium oxysporum* (strain 551), were tested against clinical isolates of *P. aeruginosa*, including a strain obtained from a burn patient. Their results showed that the bio-AgNP presented MIC values ranging from 15.62 to 62.50 μM. In another study developed by Sumini et al. (2023) [55], AgNP synthesized chemically presented a MIC of 1.5 µg/mL against *P. aeruginosa* ATCC 9027. In a study developed by da Cunha et al. (2023) [56], bio-AgNP synthesized by *Fusarium oxysporum* (strain 551) were tested against clinical isolates of *S. aureus* and MDR coagulase-negative *Staphylococcus*, and the MIC values ranged between 3.75 and 15 μg/mL, while in the study developed by Scandorieiro et al. (2016) [28], in which bio-AgNP were also produced by the fungi *F. oxysporum*, the MIC values ranged between 0.596 and 1.193 μg/mL for *S. aureus* ATCC 25923 and MRSA. In this same study, for two strains of *K. pneumoniae* (ATCC 10031 and ATCC 700603), the MIC of bio-AgNP was 0.596 μg/mL.

In this study, the MIC values for bio-AgNP ranged between 0.0053 and 0.0212 mg/mL (5.3–21.2 μg/mL). These results are close to those reported in the literature. In fact, the antimicrobial activity of AgNP is notable, with action against Gram-positive and Gram-negative bacteria, including multidrug-resistant microorganisms [28,57]. The exact antimicrobial mechanism of action of AgNP is not fully understood, however, in the literature, it is reported that the main steps include adhesion of AgNP to the bacterial surface; destabilization of the cell wall and membrane, with alteration of its permeability; induction of oxidative stress by the generation of reactive oxygen species; and modulation of signal transduction pathways [23].

Related to the OEO, in a study developed by Saoudi et al. (2024) [58], their EO was tested against reference strains of *P. aeruginosa* (ATCC 27853) and *K. pneumoniae* (ATCC 700603) and clinical isolates of *P. aeruginosa* (150) and *K. pneumoniae* (5096). The MIC values were greater for *K. pneumoniae*, with results of 2.35 and 1.2 mg/mL, than for *P. aeruginosa*, with values of 12.3 and 7.6 mg/mL (reference strain and clinical isolate, respectively). In another study, developed by Hao, Li, and Shi (2021) [59], OEO showed a MIC of 0.125 mg/mL for *S. aureus*, while in the work developed by Tejada-Muñoz et al. (2024) [60], the MIC value for a reference strain of *S. aureus* (ATCC 25923) and a clinical isolate of the same bacteria was 1.9 mg/mL. In this study, the OEO MIC values ranged between 0.59 and 9.5 mg/mL, and these results are similar to those reported in the literature.

In addition to AgNP, natural compounds, which can be derived from plants, such as essential oils, are used to combat multidrug-resistant microorganisms [61]. *Origanum vulgare* L., known as oregano, is used in cooking and as a medicinal plant. The extracted essential oil has a strong aroma, and its chemical composition includes several molecules, such as terpenes, flavonoids, and tannins, among others [62]. Given the variety of molecules present in the chemical composition of the essential oil, its antimicrobial activity can be attributed to action on different targets. These events are not isolated and occur both on the cell surface and in the cytoplasm [61]. The lipophilic nature of the essential oil allows interaction with cell membrane proteins, which alters selective permeability. With the increase in its permeability, the extravasation of ions and cellular content occurs, compromising cellular homeostasis. Subsequently, coagulation of the cytoplasm and reduction of the proton-motor force occur, which decreases ATP production [61,63]. The diversity of targets on which the essential oil acts leads to a lower chance of developing resistance compared to antibiotics [29].

Studies have shown that the combination of bio-AgNP and OEO acts synergistically against several bacterial species, including methicillin-resistant *S. aureus* (MRSA), extended-spectrum beta-lactamase (ESBL)-producing *E. coli*, and carbapenemase-producing *K. pneumoniae* (KPC) strains [28,64]. The results found in this work show that the combination of antimicrobial compounds was additive for multidrug-resistant and reference isolates of *K. pneumoniae* and *S. aureus*, in agreement with the findings of Scandorieiro et al. (2016, 2022) [28,64]. Notably, *P. aeruginosa* showed a synergistic and additive interaction, contrary to what was reported by the same authors, where the interaction was considered indifferent.

Related to the treatment of wounds caused by burns, there are several therapeutic resources available on the market, including dressings and systemic and topical treatment with antimicrobials to control infection [30,65]. The use of topical antimicrobials is recommended since the active ingredient is directly applied to the site of action, which offers advantages, such as a lower incidence of adverse effects compared to systemic administration, in addition to ease of use [15,65,66]. A form of topical treatment that can be used in burn injuries is a hydrogel due to its versatility. Furthermore, hydrogels are available in several forms, including solid, semi-solid, and liquid [67], which allows their wide application in the medical field and the incorporation of antimicrobial actives, including nanoparticles and essential oils [34].

When developing the hydrogel presented in this work, not only was the need for a product that could be easily removed during wound cleaning considered, but also the fact that the formulation would keep the area moist to promote the healing process [68,69] and would preserve the antimicrobial activity of the active ingredients. Thus, the gelling agent that met the requirements was Lecigel™. According to the technical sheet, the manufacturer describes that the synergistic combination of phospholipids and the gelling property of the copolymer results in a product with a pleasant texture and a refreshing touch. In addition to improving the penetration and bioavailability of the active ingredients, the skin’s hydration level increases [70]. The developed formulation had its hydration level improved with the addition of glycerin and propylene glycol, retaining the moisture at the wound site. Finally, the incorporation of antimicrobial actives made hydrogel a multifunctional product, preventing the development of multidrug resistance.

All hydrogels remained stable after the pre-stability test, without any sign of phase separation or alteration in the organoleptic characteristics. Related to the physical–chemical tests, the pH of all formulations was closer to 6.9, which corresponds to the pH of wounds [45], and the density was closer to 10.0, which is expected in cosmetics, where the density usually lies in a range between 9.8 and 10.0 [71]. The spreadability of hydrogels increased proportionally to the weight applied, an important parameter during the application of the formulation in burn injuries. Regarding the preliminary stability test, as shown in Table 3, the formulations did not change their initial organoleptic characteristics after fifteen days of thermal stress, except for the color of the hydrogels containing bio-AgNP, in which a small variation under conditions of temperature changes is expected and accepted [46]. The pH and density results showed few variations between T0 and T15; however, small changes are expected and do not disprove the formulation, as indicated in the Cosmetics Stability Guide [46], and considering that the hydrogel has a high-water content, the reduced density at T15 can be explained by the water evaporation.

The antimicrobial activity of hydrogels was assessed by time–kill kinetic assays, and the results are shown in Figure 2. The formulations containing bio-AgNP (HAgNP, HAgNP + OEO1, and HAgNP + OEO0.5) and SS exhibited bactericidal activity after 2 h against *P. aeruginosa* strains (1461, 1634, 117) and *K. pneumoniae* 11091. The HO0.5 hydrogel demonstrated bacteriostatic activity by maintaining a constant CFU/mL level for 24 h. For *S. aureus* 999, HAgNP + OEO1 showed bactericidal effects after 2 h, while other formulations required 8 h for the same result. HOEO1, unlike Gram-negative bacteria, showed bacteriostatic activity for *S. aureus*, while HOEO0.5 had no significant effect.

Microorganisms have different mechanisms to evade antimicrobial action; therefore, the combined use of antimicrobials is a promising strategy employed in recent years [72]. The variety of constituents with antimicrobial action present in OEO can attack different targets in the microbial cell and reduce the likelihood of developing multidrug resistance [73]. Silver nanoparticles have been used for various purposes, such as in the medical field, in textile products, and in cosmetics [74]. Despite their excellent activity against multidrug-resistant microorganisms, studies warn about the potential development of resistance to the antimicrobial action of silver nanoparticles [75,76,77]. Therefore, we highlight the relevance of the combination of bio-AgNP and OEO as a promising strategy to prevent the development of multidrug resistance.

Due to its great antimicrobial activity, the hydrogel HAgNP + OEO1 was chosen for alternative antimicrobial tests. The formulations HAgNP + OEO1 and SS were evaluated for their antimicrobial activity in an ex vivo model, while the base formulation was considered the negative control. Porcine skin has similarities to the anatomy of human skin, providing valuable features that are not present in in vitro assays [78]. The ex vivo assay model using porcine skin allows mimicking an infection with the elements present at the wound site without adding supplementary media, allowing an analysis closer to the in vivo model [40,79].

In this work, *P. aeruginosa* and *S. aureus* were used because they are microorganisms prevalent in infected wounds [80]. These bacteria are responsible for infections in lesions that can be caused by burns and pressure ulcers, among others. These infections can contribute to the complication of the patient’s clinical condition, prolonging the length of hospital stay [14,81].

Both treatments (HAgNP + OEO1 and SS) showed a decrease in the count of microorganisms on the surface and in the burn tissue (Figure 3), with variations between bacteria. On the burn surface for *S. aureus* 999, both treatments showed a decrease from log 6.0 to 4.0, indicating an approximate 99% reduction in the microbial load. A similar situation occurred with *P. aeruginosa* 1461, although the reduction was slightly smaller.

In the burn tissue, the treatment with HAgNP + OEO1 for *P. aeruginosa* 1461 showed the same reduction in the count of microorganisms as SS. This indicates that the developed hydrogel has a similar performance to the treatment used in infected wounds and burns. The treatment with HAgNP + OEO1 in *S. aureus* 999 showed a decrease from log 6.0 to 4.6, unlike the treatment with 1SS (log 6.0 to 5.33). Lecigel™ may have played an important role in the penetration and bioavailability of antimicrobial actives to the detriment of SS.

One of the challenges when using animals in experiments is the removal of the topical formulation, where the test conditions can be compromised and affect the study analysis [40]. The results of this model allow for prior evaluation and screening in the development of formulations with antimicrobial activity by mimicking in vivo conditions [50]. This model can also be used to apply other forms of treatment, such as gels, solutions, ointments, and dressings [40]. In addition to being an alternative to the use of experimental animals, the material can be obtained from a commercial source with no ethical implications.

The formulation HAgNP + OEO1 was also evaluated for its antimicrobial activity in an in vivo model with *Galeria mellonella* larvae, as shown in Figure 4. After 24 h, survival rates were 100% for the control, 90% for burn and bacteria controls, 80% for SS, and 70% for HAgNP + OEO1. After 48 h, survival was 84% for control, 80% for bacteria control, 70% for burn control, and 50% for both treatments. No significant difference was observed (*p* < 0.1386).

Studies report the use of *Galleria mellonella* as a model of infection with microorganisms isolated from multidrug-resistant clinical samples, such as *P. aeruginosa*, *S. aureus*, *K. pneumoniae*, among others [82,83]. The authors report that in 24 h, the mortality rate of larvae varied from 25% to 100% among bacterial species, with *P. aeruginosa* showing high mortality among larvae in the same period [52,82]. Maslova et al. (2023) [52] developed a model of infected burns using a clinical isolate of *P. aeruginosa*. They observed that, after 50 h, the survival rate was around 25%. Our results show that, at 48 h, the survival rate was 50%, highlighting the importance of antimicrobial treatment for larval survival. Furthermore, it is important to mention that the immune system of *Galleria mellonella* presents structural and functional similarities with the immune system of mammals [84]. Therefore, the results obtained with this invertebrate against pathogens can be comparable with the response observed in mammals [85,86].

*Galleria mellonella* has been used in assays to evaluate the antimicrobial efficacy of natural components and antimicrobial combinations [85]. The concept of the ‘3 Rs’ (replace, reduce, and refine) is applicable in this work by replacing the use of mammals in infection assays. It is worth noting that this invertebrate is not subject to restrictions under animal welfare legislation [37,83].

For the first time, the combination of bio-AgNP and OEO was incorporated into a formulation for wounds’ burn treatment, and the results demonstrated a great potential of this hydrogel to be an alternative treatment to the conventional options available in the market. The focus of this work was testing the hydrogel with antimicrobials against multidrug-resistant bacteria using alternative ex vivo and in vivo methods. Therefore, a limitation of this work was the lack of in vivo testing in murine models, which will be carried out in future studies, as well as clinical tests in burn patients.

## 5. Conclusions

The combination of bio-AgNP and OEO demonstrated bactericidal activity against reference strains and clinical isolates of multidrug-resistant Gram-negative and Gram-positive species, being a potential antimicrobial alternative for the treatment of infected wounds caused by burns. The hydrogel containing both actives remained stable after the pharmacotechnical tests and showed great antimicrobial activity, similar to the conventional treatment with silver sulfadiazine, which was demonstrated through specific and alternative burn models, in substitution to in vivo models. This product has great market potential, and due to the growing concern about multidrug-resistant microorganisms, it is necessary to search for alternatives to prevent infections in burns. Furthermore, this work highlights the importance of using alternative methods to evaluate antimicrobial action in burn injuries. Further research is needed to demonstrate the antimicrobial activity of this hydrogel through clinical trials in burn patients and its possible effects, such as moisture property.

## Figures and Tables

**Table 1:** Origin and sensibility of the clinical isolates to antimicrobials.

Microorganism	Origin	ID	Resistance Profile to Antimicrobials	
*Klebsiella* *pneumoniae*	Tissue	10904	PPT, FEP, CTX, CPD, CAZ, CRO, CRX, LEV, CIP, TET, GEN, TOB, ETP, IPM, MEM, SUT, CLO	KPC MDR
	Tissue	11034	PPT, FEP, CTX, CPD, CAZ, CRO, CRX, LEV, CIP, TET, GEN, TOB, ETP, IPM, MEM, SUT, AMI, CLO	KPC MDR
	Tissue	11091	PPT, FEP, CTX, CPD, CAZ, CRO, CRX, LEV *, CIP, TET, GEN, TOB, ETP, IPM, MEM, SUT, CLO *	KPC MDR
	Tissue	11271	PPT, FEP, CTX, CPD, CAZ, CRO, CRX, LEV, CIP, TET, GEN, TOB, ETP, IPM, MEM, AZI, SUT, CLO	KPC MDR
	Bone Fragment	10797	PPT, FEP, CTX, CPD, CAZ, CRO, CRX, LEV, CIP, TET, GEN, TOB, ETP, MEM, AZI, SUT, CLO	KPC MDR
*Pseudomonas* *aeruginosa*	Urine	2491	LEV, CAZ, CIP, TOB, ATM, PPT, IPM, MEM	CR MDR
	Tissue	3167	LEV, CAZ, CIP, TOB, ATM, PPT, IPM, MEM	CR MDR
	Tissue	1461	LEV, CAZ, CIP, TOB, ATM, PPT, IPM, MEM	CR MDR
	Tissue	2815	LEV, CAZ, CIP, TOB, ATM, PPT, IPM, MEM	CR MDR
	Urine	117	LEV, TGC, CAZ, CIP, ATM, PPT, IPM, MEM, GEN, NOR, NAL	CR MDR
	Human patient	1634	LEV, TGC, CAZ, CIP, TOB, ATM, PPT, IPM, MEM, GEN, NOR, NAL	CR MDR
*Staphylococcus* *aureus*	Tissue	373	P, OX, AZI, CIP, CLO *, CLI, ERI, QD	MRSA
	Tissue	207	P, OX, AZI, CIP, CLO *, CLI, ERI, QD	MRSA
	Tissue	521	P, OX, AZI, CIP, CLI, ERI, QD	MRSA
	Tissue	999	P, OX, AZI, CIP *, CLI, ERI, QD	MRSA
	Tissue	336	P, OX, AZI, CIP, SUL, CLI, ERI, QD	MRSA

ID: identification; * interpretation of the inhibition zone diameter: intermediate; Antimicrobials: clindamycin (CLI), ciprofloxacin (CIP), levofloxacin (LEV), rifampin (RD), cefpodoxime (CPD), tobramycin (TOB), ertapenem (ETP), imipenem (IPM), meropenem (MEM), gentamicin (GEN), azithromycin (AZI), quinupristin-dalfopristin (QD), erythromycin (ERI), ampicillin-sulbactam (SAM), cefepime (FEP), cefotaxime (CTX), ceftazidime (CAZ), ceftriaxone (CRO), cefuroxime (CRX), cefoxitin (CFO), tetracycline (TET), linezolid (LZD), amoxicillin-clavulanic acid (AMC), chloramphenicol (CLO), trimethoprim-sulfamethoxazole (SUT), and piperacillin-tazobactam (PPT), amikacin (AMI), aztreonam (ATM), tigecyclin (TGC), norfloxacin (NOR), nalidixic acid (NAL), penicillin (P), oxacillin (OX), sulfonamide (SUL).

**Table 2:** Antimicrobial activity of the actives against the clinical isolates.

	Bio-AgNP (mg/mL)			OEO (mg/mL)			FICI	Interaction
	MIC_ind_	MIC_comb_	FIC	MIC_ind_	MIC_comb_	FIC		
*P. aeruginosa* 1461	0.0212	0.0026	0.12	4.75	1.18	0.25	0.37	Sinergism
*P. aeruginosa* ATCC 27853	0.0053	0.0013	0.25	2.37	0.59	0.25	0.49	Sinergism
*P. aeruginosa* 2491	0.0106	0.0013	0.25	4.75	1.18	0.13	0.38	Aditism
*P. aeruginosa* 2815	0.0106	0.0013	0.12	9.50	2.37	0.25	0.37	Sinergism
*P. aeruginosa* 117	0.0106	0.001	0.09	9.50	1.18	0.12	0.22	Sinergism
*P. aeruginosa* 1634	0.0106	0.001	0.09	9.50	1.18	0.12	0.22	Sinergism
PRSA 999	0.0106	0.0026	0.24	0.59	0.296	0.50	0.74	Aditism
PRSA 207	0.0212	0.0026	0.12	1.18	0.59	0.50	0.62	Aditism
PRSA 521	0.0212	0.0106	0.47	1.18	0.29	0.25	0.72	Aditism
*S.aureus* ATCC 6538	0.0212	0.0026	0.12	1.18	0.59	0.50	0.62	Aditism
MRSA N315	0.0212	0.0212	0.10	1.18	1.18	0.50	0.47	Aditism
*K.pneumoniae* 11034	0.0212	0.0026	0.12	1.18	0.59	0.50	0.62	Aditism
*K.pneumoniae* 10797	0.0212	0.0106	0.47	0.59	0.07	0.13	0.60	Aditism
*K.pneumoniae* ATCC 700603	0.0212	0.0026	0.12	1.18	0.59	0.50	0.62	Aditism

Bio-AgNP: biogenic silver nanoparticles; OEO: *Origanum vulgare* essential oil; MIC_ind_: minimum inhibitory concentration of the actives isolate; MIC_comb_: minimum inhibitory concentration of the actives in combination; FIC: Fractional Inhibitory Concentration; FICI: Fractional Inhibitory Concentration Index; PRSA: *S. aureus* penicillin-sensitive; MRSA: *S. aureus* methicillin-resistant.

**Table 3:** Organoleptic characteristics of hydrogels at the preliminary stability test.

	Color		Odor		Aspect	
Formulation	T0	T15	T0	T15	T0	T15
Base	White	N	Odorless	N	Homogeneous	N
HAgNP	Caramel	LM	Odorless	N	Homogeneous	N
HO1	White	N	OEO	N	Homogeneous	N
HO0.5	White	N	OEO	N	Homogeneous	N
HagNP + OEO1	Caramel	LM	OEO	N	Homogeneous	N
HagNP + OEO0.5	Caramel	LM	OEO	N	Homogeneous	N

T0: time zero; T15: after 15 days of thermal stress; OEO: odor characteristic to OEO; N: normal; LM: slightly modified. Base: formulation without actives; HAgNP: hydrogel containing bio-AgNP 500 µM; HO1: hydrogel containing OEO 1%; HO0.5: hydrogel containing 0.5% OEO; HAgNP + OEO1: hydrogel containing bio-AgNP 500 µM + OEO 1%; HAgNP + OEO0.5: hydrogel containing bio-AgNP 500 µM + OEO 0.5%.

**Table 4:** pH and density of hydrogels at the preliminary stability test.

	pH		Density	
Formulation	T0	T15	T0	T15
Base	6.95 ± 0.03	6.83 ± 0.02	9.95 ± 0.01	9.91 ± 0.02
HAgNP	6.94 ± 0.02	6.53 ± 0.02	10.10 ± 0.02	9.99 ± 0.01
HO1	6.92 ± 0.04	6.57 ± 0.01	10.06 ± 0.02	9.96 ± 0.02
HO0.5	6.96 ± 0.02	6.59 ± 0.02	10.05 ± 0.02	9.99 ± 0.01
HagNP + OEO1	6.94 ± 0.04	6.56 ± 0.03	10.14 ± 0.03	9.99 ± 0.01
HagNP + OEO0.5	6.97 ± 0.03	6.87 ± 0.02	10.10 ± 0.02	10.02 ± 0.01

T0: time zero; T15: after 15 days of thermal stress. Base: formulation without actives; HAgNP: hydrogel containing bio-AgNP 500 µM; HO1: hydrogel containing OEO 1%; HO0.5: hydrogel containing 0.5% OEO; HAgNP + OEO1: hydrogel containing bio-AgNP 500 µM + OEO 1%; HAgNP + OEO0.5: hydrogel containing bio-AgNP 500 µM + OEO 0.5%.

## Data Availability

The original contributions presented in the study are included in the article; further inquiries can be directed to the corresponding author.
